# Human Heart–Macrophage Assembloids Capture Tissue-Resident Immune Integration During Cardiac Development

**DOI:** 10.51894/001c.163998

**Published:** 2026-07-31

**Authors:** Freyda Mannering, Colin O’Hern, Sammantha Caywood, Shakhlo Aminova, Artem Kiselev, Brett Volmert, Weiheng Cao, Fei Wang, Mia Dionise, Merlinda-Loriane Sewavi, Milana Skoric, Hussain Basrai, Priyadharshni Muniyandi, Mirel Popa, George Boulos, Kyle Wolf, Izabelle Brown, Isabel Nuñez-Regueiro, Amanda Huang, Aleksandra Kostina, Lauren Squire, Curtis Wilkerson, Nagib Chalfoun, Sangbum Park, Nureddin Ashammakhi, Chao Zhou, Christopher Contag, Aitor Aguirre

**Affiliations:** 1 College of Osteopathic Medicine, Michigan State University

## 05

### Objectives

Here, we describe the engineering and systematic characterization of human heart–macrophage assembloids (hHMA) that recapitulate key features of cardiac immune–parenchymal interactions.

### Methods

Human pluripotent stem cells were differentiated into cardiogenic organoids using staged Wnt modulation, while macrophages were generated in parallel via myeloid differentiation and introduced during defined windows of organoid development. Integrated macrophages stably persisted within the three-dimensional tissue, localized to cardiomyocyte-rich regions and extracellular matrix interfaces, and exhibited marker expression consistent with tissue-resident macrophage–like states rather than acute inflammatory phenotypes. Specific methods such as immunohistochemistry, brightfield and confocal microscopy, live imaging, flow cytometry, optical coherence tomography, singe-cell transcriptomics, and proteomic analysis were used to derive these conclusions.

### Results

Structural and molecular analyses revealed that hHMA displayed phenotypic features consistent with human embryonic cardiac tissue-resident macrophage–like states, and were spatially integrated throughout the organoid interior. At the tissue level, OCT and structural analyses showed that hHMAs retained organized 3D architecture, while multiomic profiling demonstrated that macrophage incorporation reshaped paracrine signaling, supported efferocytotic programs, and altered extracellular matrix remodeling and electrical-conduction-associated pathways.

### Discussion

Together, these data establish a reproducible human heart–immune assembloid platform that captures stable macrophage integration within cardiac tissue. This system provides a versatile foundation for interrogating immune contributions to human cardiac development, maturation, and disease beyond what is achievable with immune-naïve heart organoids.

